# T-cell-targeted immunotherapy in neurofibromatosis type 2-related vestibular schwannoma: current evidence and future direction

**DOI:** 10.1093/braincomms/fcag192

**Published:** 2026-05-28

**Authors:** Reygn J Done, Omar Ahmid, Miriam J Smith, D Gareth Evans, Omar N Pathmanaban, David Brough, Kevin N Couper

**Affiliations:** Lydia Becker Institute of Immunology and Inflammation, Division of Immunology, Immunity and Infection & Respiratory Medicine, Faculty of Biology, Medicine & Health, The University of Manchester, Manchester M13 9PT, UK; Geoffrey Jefferson Brain Research Centre, Manchester Academic Health Science Centre, Northern Care Alliance NHS Foundation Trust, University of Manchester, Manchester M13 9PT, UK; Geoffrey Jefferson Brain Research Centre, Manchester Academic Health Science Centre, Northern Care Alliance NHS Foundation Trust, University of Manchester, Manchester M13 9PT, UK; Division of Neuroscience, Faculty of Biology, Medicine & Health, The University of Manchester, Manchester M13 9PT, UK; Geoffrey Jefferson Brain Research Centre, Manchester Academic Health Science Centre, Northern Care Alliance NHS Foundation Trust, University of Manchester, Manchester M13 9PT, UK; Manchester Centre for Genomic Medicine, St Mary’s Hospital, Division of Evolution, Infection and Genomics, Faculty of Biology, Medicine & Health, The University of Manchester, Manchester M13 9PT, UK; Geoffrey Jefferson Brain Research Centre, Manchester Academic Health Science Centre, Northern Care Alliance NHS Foundation Trust, University of Manchester, Manchester M13 9PT, UK; Manchester Centre for Genomic Medicine, St Mary’s Hospital, Division of Evolution, Infection and Genomics, Faculty of Biology, Medicine & Health, The University of Manchester, Manchester M13 9PT, UK; Geoffrey Jefferson Brain Research Centre, Manchester Academic Health Science Centre, Northern Care Alliance NHS Foundation Trust, University of Manchester, Manchester M13 9PT, UK; Division of Neuroscience, Faculty of Biology, Medicine & Health, The University of Manchester, Manchester M13 9PT, UK; Department of Neurosurgery, Manchester Centre for Clinical Neurosciences, Salford Royal Hospital, Northern Care Alliance NHS Foundation Trust, Salford M6 8HD, UK; Geoffrey Jefferson Brain Research Centre, Manchester Academic Health Science Centre, Northern Care Alliance NHS Foundation Trust, University of Manchester, Manchester M13 9PT, UK; Division of Neuroscience, Faculty of Biology, Medicine & Health, The University of Manchester, Manchester M13 9PT, UK; Lydia Becker Institute of Immunology and Inflammation, Division of Immunology, Immunity and Infection & Respiratory Medicine, Faculty of Biology, Medicine & Health, The University of Manchester, Manchester M13 9PT, UK; Geoffrey Jefferson Brain Research Centre, Manchester Academic Health Science Centre, Northern Care Alliance NHS Foundation Trust, University of Manchester, Manchester M13 9PT, UK

**Keywords:** vestibular schwannoma, neurofibromatosis type 2-related schwannomatosis, T-cell, immunotherapy, immune

## Abstract

Neurofibromatosis type 2-related schwannomatosis is a rare tumour predisposition syndrome caused by loss-of-function pathogenic variants within the *NF2* gene, which encodes the tumour suppressor protein merlin. This leads to development of benign tumours within the nervous system, most notably vestibular schwannomas that form on the vestibulocochlear (eighth cranial) nerve. Whilst the merlin-regulated molecular mechanisms in neoplastic Schwann cells that underlie vestibular schwannoma formation and growth are increasingly understood, the role of the tumour-immune microenvironment—particularly T-cells—in influencing vestibular schwannoma pathology and the clinical trajectory of disease remain understudied. In this review, we outline fundamental principles of T-cell immunobiology and anti-tumour immunity and examine their relevance to neurofibromatosis type-2-related schwannomatosis-associated vestibular schwannoma. We describe how spatial and transcriptomic profiling of tumour-infiltrating lymphocytes have revealed hallmarks of T-cell exhaustion and immunoevasion within vestibular schwannoma tumours and the existence of myriad immunosuppressive mechanisms within the vestibular schwannoma microenvironment—including immune checkpoint ligand expression, immunosuppressive cytokines, regulatory immune subsets and metabolic constraints. We also review the key remaining biological prerequisites, such as T-cell dynamics, T-cell functional state and T-cell spatial context within the tumour microenvironment, that must be addressed before advanced T-cell-based strategies can be rationally developed for vestibular schwannoma.

## Teaser

Done *et al*. review the biology of neurofibromatosis type-2-associated vestibular schwannomas, focusing on current immunological understanding of the tumour microenvironment. They contextualize the immunological basis of T-cell-targeted therapy to support the rational development of new immunotherapeutic strategies for neurofibromatosis type-2-associated vestibular schwannoma.

## Graphical Abstract

**Graphical Abstract:**
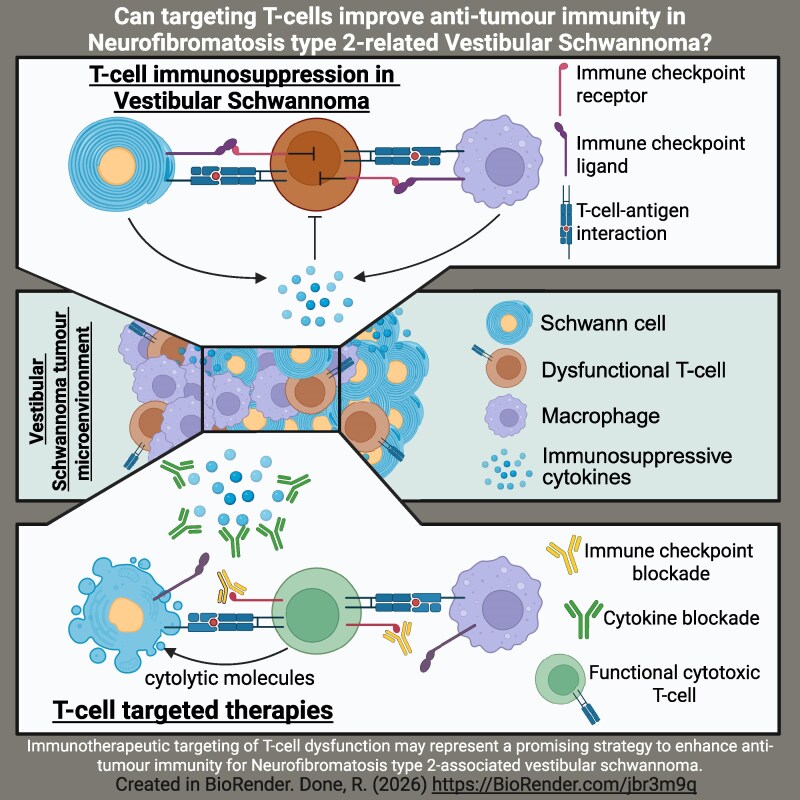

## Introduction

Neurofibromatosis type 2*-*related schwannomatosis (*NF2-*SWN) is a rare neurocutaneous tumour predisposition disorder, characterized by the formation of benign tumours within the CNS and peripheral nervous system (PNS), most notably vestibular schwannoma (VS).^1^ The condition was historically referred to as neurofibromatosis type 2, which grouped it alongside other genetically distinct neurocutaneous syndromes, including neurofibromatosis type 1 (NF1) and schwannomatosis. However, consensus recommendations have recently revised the nomenclature to *NF2*-SWN, reflecting the underlying molecular aetiology and recognizing that neurofibromas are not a feature of this condition.^2^  *NF2*-SWN is now classified within the broader spectrum of schwannomatosis, which comprise genetically distinct disorders characterized by the development of multiple schwannomas. Distinguishing *NF2*-SWN from other forms of schwannomatosis (*SMARCB1*- or *LZTR1*-related schwannomatosis) can be clinically challenging, particularly in individuals presenting without bilateral VS, thereby underscoring the importance of molecular genetic testing for accurate diagnoses.^2^ Key clinical and molecular differences between *NF2-*SWN and Schwannomatosis are summarized in Table 1.

**Table 1:** Comparison of neurofibromatosis type 2-related schwannomatosis and schwannomatosis

Feature	Neurofibromatosis type 2-related schwannomatosis	Schwannomatosis
Causal gene mutants	*NF2*	*SMARCB1*, *LZTR1*
Encoded protein	Merlin	SMARCB1, LZRT1
Major tumour types	Bilateral vestibular schwannoma	Multiple painful schwannomas
Location	Cranial, peripheral and spinal nerves, meninges	Peripheral nerves
Cell origin	Schwann cells and arachnoid cap cells	Schwann cells
Primary signalling pathways affected	Hippo, PI3K, AKT-mTOR	Variable; multiple tumour pathways
Epidemiology	1/33 000 births	1/40 000 births

*NF2*-SWN results from autosomal dominant pathogenic variants within the *NF2* gene located on chromosome 22q12, which encodes the tumour-suppressor protein merlin (schwannomin). Constitutional inactivation of merlin disrupts normal Schwann cell function, predisposing individuals to bilateral VS arising on the vestibulocochlear nerve (eighth cranial nerve).^3^ Although histologically benign, VS are a major cause of morbidity, frequently causing progressive hearing loss, tinnitus, balance dysfunction and facial nerve deficits.

*NF2*-SWN has an estimated birth incidence and diagnostic prevalence of ∼1/60 000 worldwide.^4^ Current research continues to focus on identifying potential therapeutic targets to improve treatment options for those with *NF2*-SWN; however, management remains challenging and is often associated with incomplete tumour control, adverse effects and variable, long-term outcomes.^5^ Emerging evidence suggests that the VS tumour microenvironment (TME) may be amenable to immunomodulation, raising the possibility that T-cell-targeted immunotherapies could provide a novel therapeutic avenue. Accordingly, this review focusses on the potential of T-cell-targeted immunotherapy in *NF2-*SWN, with a particular emphasis on VS given their substantial contribution to disease burden and quality-of-life impairment. In addition, the review outlines fundamental principles of T-cell immunobiology and anti-tumour immunity to provide a conceptual framework for understanding how T-cell responses could be effectively leveraged as therapeutic strategies for *NF2*-SWN-associated VS.

## *NF2*-SWN-associated tumours

VS, arising within the cerebellopontine angle (CPA), are a defining characteristic and pathognomonic feature of *NF2*-SWN, making up ∼75% of tumours in this region.^6^ Whilst 95% of VS cases are sporadic and unilateral, the remaining 5% are bilateral and are primarily associated *NF2*-SWN.^7^ Notably, ∼60–70% of sporadic VS tumours also exhibit biallelic somatic *NF2* gene mutations/chromosomal loss,^8^ emphasizing the importance of loss of merlin function in promoting VS tumour development.^9^ VS tumours, although benign, significantly impair quality of life by the tumours, or their treatments, causing deafness, facial nerve paralysis and ocular abnormalities, with severe cases leading to hydrocephalus and brainstem compression, which can be fatal.^10,11^

**Figure 1:**
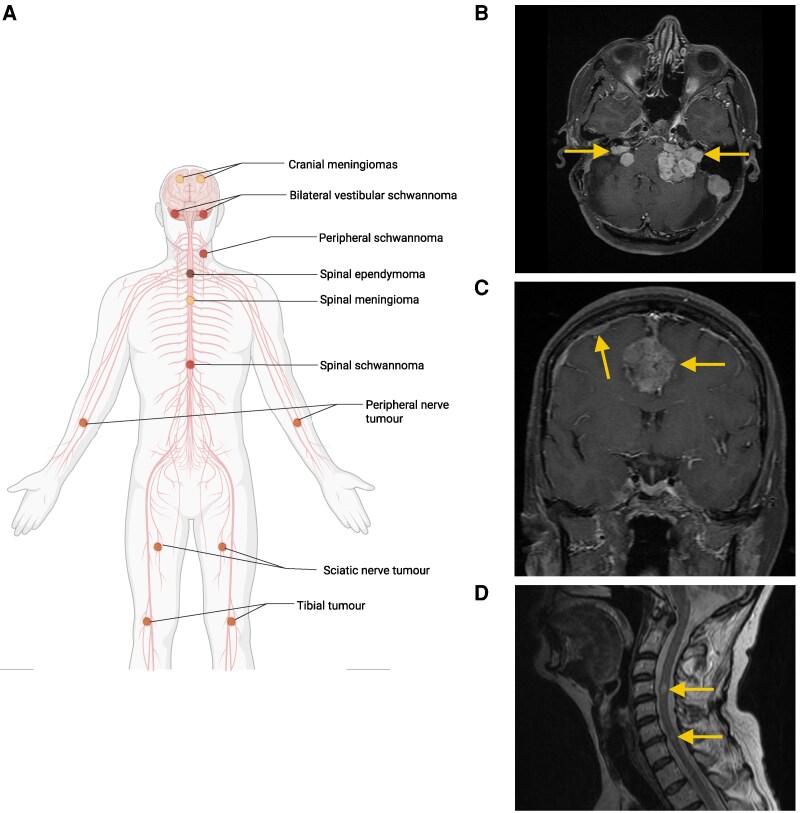
**Common tumour locations in neurofibromatosis type 2-related schwannomatosis.** This schematic illustrates (**A**) the typical anatomical regions where tumours commonly develop in individuals with neurofibromatosis type 2-related schwannomatosis, including cranial nerves spinal cord and peripheral nerves. The most frequently affected site includes the vestibulocochlear nerve. (**B**) Axial T1 with contrast MRI of bilateral vestibular schwannomas indicated by yellow arrows. (**C**) Coronal T1 with contrast MRI of multiple meningiomas indicated by yellow arrows. (**D**) Sagittal T2 MRI of spinal cord ependymoma indicated by yellow arrows. (**A**) Created in BioRender. Ahmid, O. (2026), https://BioRender.com/i043l64.

*NF2*-SWN is also associated with spinal, cranial and peripheral nerve tumours, as depicted in Fig. 1. Seventy per cent of *NF2*-SWN patients develop peripheral plexiform schwannomas originating from the dorsal root.^12^ The condition is further complicated by meningiomas, which are found in up to 58% of patients, and is the second most common tumour type in *NF2*-SWN patients.^13^ Furthermore, these *NF2*-mutant supratentorial meningiomas are mostly world-health organization grade II or III and are associated with higher morbidity, seizures and potential for brainstem compression.^14-17^ Although meningiomas constitute a clinically important component of the *NF2*-SWN tumour spectrum, the primary focus of this review is on NF2-SWN-associated VS. We refer readers to a recent separate review for detailed coverage of *NF2*-SWN-associated meningiomas and other extra-axial brain tumours,^18^ including in the context of the skull-meninges-brain immune axis.

Moreover, ependymomas form in up to ∼50% of *NF2*-SWN patients^13^ and arise from ependymal cells, although typically without clinical symptoms.^19^ Thus, the existence of various tumour types further complicates treatment strategies for those with *NF2*-SWN and identifying therapies that either target one tumour-type alone, or multiple of these tumour-types, is of great interest.

## Pathogenic variants in *NF2*-SWN

Following the advent of genomic technologies, accurate mapping of the *NF2* gene mutational landscape has helped resolve the variability in clinical features of *NF2*-SWN, as depicted in the genetic severity score (GSS) outlined in Table 2. This provides a stable framework for predicting clinical outcomes in *NF2*-SWN patients.^20-22^ The GSS stratifies *NF2*-SWN severity into three primary categories: tissue mosaicism (class 1A and 1B), classical (classes 2A mild and 2B moderate) and severe (class 3).

**Table 2:** *NF2* gene mutations and severity of disease^20-22^

Mutation type	Location	*NF2* severity for mosaic events (Catasus *et al.*, 2022)	*NF2* severity score for mosaic events (Halliday *et al.*, 2017)	*NF2* severity for heterozygous variants (Catasus *et al.*, 2022)	*NF2* severity for heterozygous variants (Halliday *et al.*, 2017)
Missense variant		Very mild	Mild (2A)	Mild	Mild (2A)
Deletion	Small in-frame deletion or duplication	Very mild	Mild (2A)	Mild	Mild (2A)
	Large deletions (more than 1 exon) including promotor or exon 1:				
	Maintained reading frame	Very mild	Mild (2A)	Mild	Mild (2A)
	Frameshift alteration	Mild	Mild (2A)	Moderate	Mild (2A)
	Whole NF2 gene	Mild	Mild (2A)	Moderate	Mild (2A)
	Large deletions (more than exon 1) excluding promotor or exon 1:				
	Maintaining reading frame	Very mild	Mild (2A)	Mild	Moderate (2B)
	Frameshift alteration	Mild	Mild (2A)	Moderate	Moderate (2B)
Splice site	Exons 1–7 in-frame	Mild	Mild (2A)	Moderate	Moderate (2B)
	Exons 1–7 frameshift	Moderate	Mild (2A)	Moderate > severe	Moderate (2B)
	Exons 8–15 in-frame	Mild	Mild (2A)	Moderate	Mild (2A)
	Exons 8–15 frameshift	moderate	Mild (2A)	Moderate > severe	Mild (2A)
Truncating	Exon 1	Mild > moderate	Mild (2A)	Moderate	Mild (2A)
	Exon 2–13	Moderate	Moderate (2B)	Severe	Severe (3)
	Exons 14–15	Moderate	Mild (2A)	Moderate > severe	Moderate (2B)
Tissue mosaic variant (not seen in blood)		Presumed		Proven	
		Mild (1A)		Mild (2B)	

Tissue mosaicism, found in ∼33–60% of *de novo NF2*-SWN patients, results from pathogenic *de novo* mutations arising post-zygotically.^23,24^ Interestingly, over 70% of *NF2*-SWN cases also result from *de novo* mutations.^4,24^ Typically, patients with mosaicism generally present with milder symptom presentation, but this largely depends on the mutational variant acquired and whether it is detectable in blood.^21,23,24^ Diagnosing mosaicism is challenging as it often involves low mutational burdens that many current tests miss, detecting only 15% of all mutations.^24,25^ Furthermore, individuals with *NF2* mosaicism exhibit a reduced risk of transmitting mutant *NF2* genes to offspring; however, when this happens, children of *NF2*-mosaic parents who inherit the parental *NF2* pathogenic variant will carry the variant in all cells, exhibiting a severe clinical phenotype.^25^

Nonsense and frameshift variants are amongst the most clinically significant and are associated with poorer prognoses. Both types of variants introduce premature stop codons which truncate merlin. Such truncating protein variants are usually predicted to trigger nonsense-mediated mRNA decay, resulting in complete loss of merlin expression, thus the loss of its tumour-suppressor function. Specific splice-site variants can also have truncating effects if they cause exon skipping or frameshifts.^1^ Truncating variants in exons 2–13 typically result in more severe clinical symptoms, poorer prognoses compared to mutations in exons 1, 14 and 15,^21^ and also result in increased risk of meningioma development.^26^ This link between mutational variability and clinical severity is mirrored in animal studies whereby exon 2–13 deletion promotes rapid schwannoma development.^27^ Splice-site variants at intron-exon boundaries can also cause disruptions in RNA splicing. This promotes the inappropriate exclusion and/or inclusion of incorrect exons.^28^ Such splice-site variants in exons 1–7 generally cause more severe clinical symptoms than those affecting exons 11–15.^29^

## Structure and tumour suppressing functions of merlin

The *NF2* gene encodes multiple merlin isoforms via alternative splicing, with isoforms 1 and 2 being the most functionally significant. Isoform 1, encoded by the MANE Select transcript (NM_000268.4), is particularly noted for their prominent tumour-suppressive activity.^30^ Merlin is part of the ERM (ezrin, radixin, moesin) protein family, all of which share vast structural homology, specifically within the N-terminus four-point-one ERM (FERM) domain.^31-33^ All ERM proteins function as cross-linkers between the plasma membrane and cytoskeleton, with loss of function mutations promoting disruption in cellular structure.^34,35^

**Figure 2:**
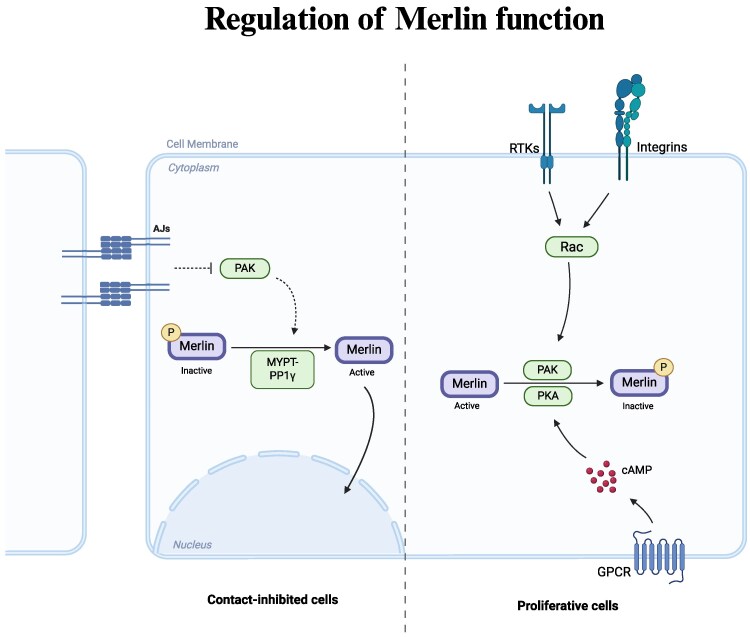
**Functional regulation of Merlin activity. Merlin activity is regulated by phosphorylation-dependent conformational changes.** In contact-inhibited cells, Merlin is maintained in an active, growth-suppressive state through dephosphorylation by MYPT–PP1γ, allowing Merlin to associate with adherens junctions and suppress proliferative signalling. In proliferative cells, upstream inputs from RTKs, integrins and GPCR/cAMP signalling activate Rac, PAK and PKA, which promote Merlin phosphorylation and inactivation. Phosphorylated Merlin adopts an inactive conformation, reducing its tumour-suppressive function and permitting downstream proliferative signalling. AJs, adherens junctions; cAMP, cyclic adenosine monophosphate; GPCR, G protein-coupled receptor; MYPT–PP1γ, myosin phosphatase targeting subunit–protein phosphatase 1 gamma; P, phosphorylation; PAK, p21-activated kinase; PKA, protein kinase A; Rac, Ras-related C3 botulinum toxin substrate; RTKs, receptor tyrosine kinases. Solid arrows indicate activation pathways, and dotted arrows/lines represent inhibitory effects. Created in BioRender. Done, R. (2026), https://BioRender.com/x1ss52n.

Structurally, merlin is composed of three major domains: (i) a membrane-binding N-terminus FERM domain,^36^ (ii) C-terminal ERM-associated domain crucial for post-translational modifications and regulation of merlin function^36,37^ and (iii) an a-helical self-regulation domain which links the C- and N-termini.^38^ Notably, phosphorylation levels of Serine-518 within the C-terminal region vary with cell density and influences cellular proliferation; hypophosphorylation of Serine-518 at high cell densities results in growth inhibition, whereas hyperphosphorylation at low densities promotes growth and potential tumour progression (Fig. 2).^39,40^ Merlin is also implicated in contact inhibition, where it regulates Rac1, a component that lies upstream of signalling components that control the phosphorylation state and conformation of merlin.^41-43^ Merlin directly and indirectly regulates multiple different signalling cascades within cells that control cellular survival, proliferation, migration and cellular metabolism.^36^ Some of these pathways are summarized here.

### Receptor tyrosine kinase function

**Figure 3:**
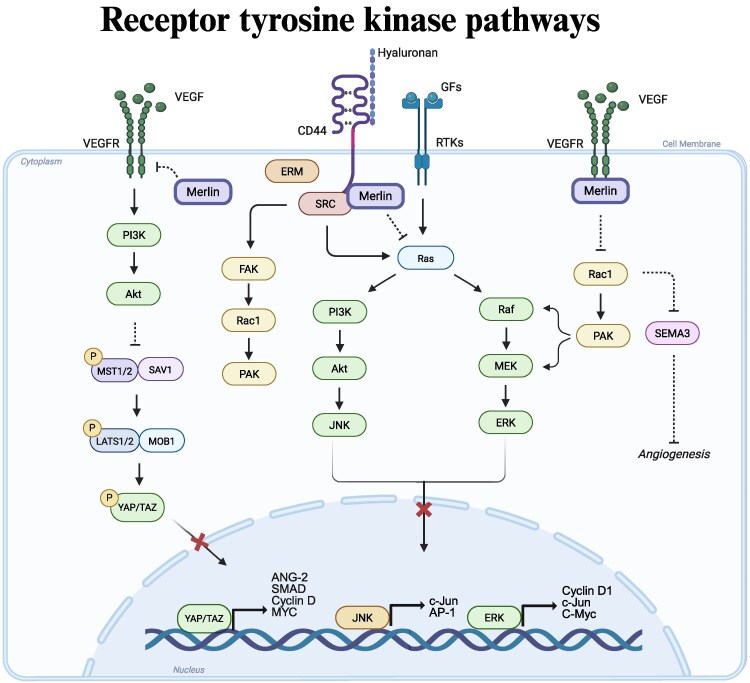
**Role of Merlin in receptor tyrosine kinase pathways.** Merlin negatively regulates multiple receptor tyrosine kinase-driven signalling pathways involved in proliferation, survival and angiogenesis. Growth factor, VEGF, CD44–hyaluronan and integrin-associated signalling converge on downstream pathways including PI3K–Akt, Ras–Raf–MEK–ERK, Rac1–PAK and JNK. Merlin restrains these pathways at multiple levels, thereby limiting activation of transcriptional programmes involving YAP/TAZ, AP-1, c-Jun, MYC and Cyclin D1. Loss or inactivation of Merlin can therefore enhance mitogenic, survival and angiogenic signalling, contributing to tumour growth. ANG-2, angiopoietin-2; AP-1, activator protein 1; CD44, cluster of differentiation 44; ERK, extracellular signal-regulated kinase; ERM, ezrin/radixin/moesin; FAK, focal adhesion kinase; GFs, growth factors; JNK, c-Jun N-terminal kinase; LATS1/2, large tumour suppressor kinase 1/2; MEK, mitogen-activated protein kinase kinase; MOB1, MOB kinase activator 1; MST1/2, mammalian STE20-like protein kinase 1/2; MYC, MYC proto-oncogene; P, phosphorylation; PAK, p21-activated kinase; PI3K, phosphoinositide 3-kinase; Rac1, Ras-related C3 botulinum toxin substrate 1; Raf, rapidly accelerated fibrosarcoma kinase; Ras, rat sarcoma virus GTPase; RTKs, receptor tyrosine kinases; SAV1, salvador family WW domain-containing protein 1; SEMA3, semaphorin 3; SMAD, mothers against decapentaplegic homologues; SRC, proto-oncogene tyrosine-protein kinase Src; VEGF, vascular endothelial growth factor; VEGFR, vascular endothelial growth factor receptor; YAP/TAZ, yes-associated protein/transcriptional coactivator with PDZ-binding motif. Solid arrows indicate activation pathways, and dotted arrows/lines represent inhibitory effects. Created in BioRender. Done, R. (2026), https://BioRender.com/0rdouuu.

Receptor tyrosine kinase’s (RTK’s), such as the platelet derived growth factor receptor (PDGFR)^44^ and epidermal growth factor receptor (EGFR) families,^45^ play major roles in controlling cellular survival and proliferation and are often upregulated in *NF2*-mutated schwannomas and *NF2*-deficient Schwann cells, respectively. Merlin’s function involves stabilizing cell structures which prevent the activation of these RTK’s (Fig. 3), thereby controlling downstream signalling pathways that typically enhance tumour growth.^46,47^ In *NF2-*mutated schwannomas, the loss of merlin leads to an overactivation of MAPK and PI3K signalling pathways. This is largely driven by dysregulated RTK’s, such as PDGFR, which normally activate these cascades. Forced expression of functional *NF2* is sufficient to reverse this effect, likely by promoting RTK internalization and degradation.^44,48^

### Mammalian target of rapamycin

**Figure 4:**
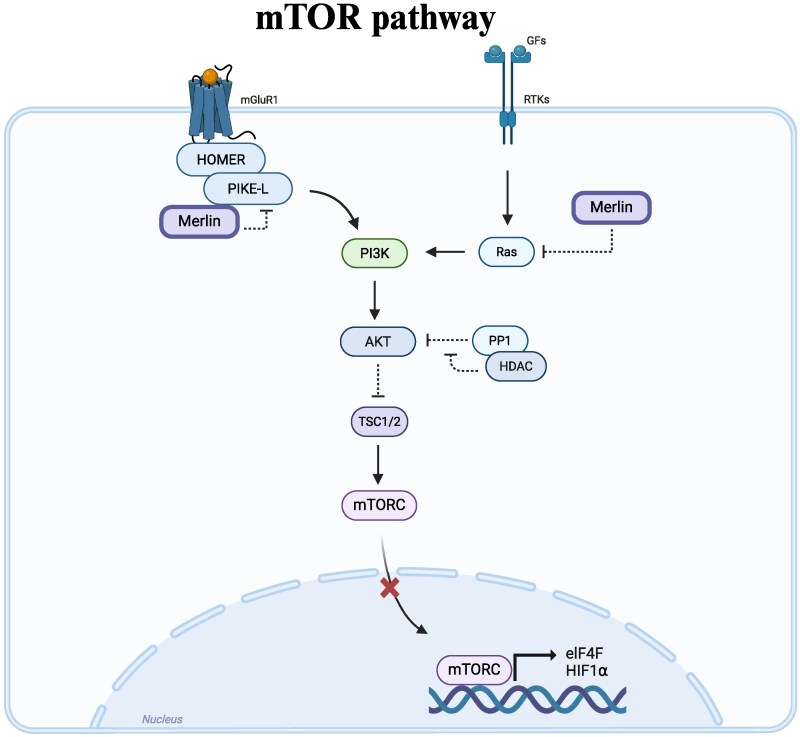
**Role of Merlin in mTOR signalling.** Merlin regulates mTOR pathway activation through receptor tyrosine kinase, Ras and PI3K–Akt signalling, as well as through mGluR1–HOMER–PIKE-L-associated inputs. Under normal conditions, Merlin suppresses upstream signalling that activates PI3K and Akt, thereby limiting downstream mTORC activation. Merlin also interacts with regulatory components such as PP1 and HDAC-associated pathways. Loss of Merlin function can enhance mTORC activity and promote transcriptional and translational programmes involving HIF1α and eIF4F, supporting cell growth and tumour progression. AKT, protein kinase B; eIF4F, eukaryotic initiation factor 4F; GFs, growth factors; HDAC, histone deacetylase; HIF1α, hypoxia-inducible factor 1-alpha; HOMER, Homer scaffold protein; mGluR1, metabotropic glutamate receptor 1; mTOR, mechanistic target of rapamycin; mTORC, mechanistic target of rapamycin complex; PI3K, phosphoinositide 3-kinase; PIKE-L, phosphoinositide 3-kinase enhancer long isoform; PP1, protein phosphatase 1; Ras, rat sarcoma virus GTPase; RTKs, receptor tyrosine kinases; TSC1/2, tuberous sclerosis complex 1/2. Solid arrows indicate activation pathways, dotted arrows/lines represent inhibitory effects. Created in BioRender. Done, R. (2026), https://BioRender.com/1br0n6b.

The mammalian target of rapamycin (mTOR) pathway is essential for cell growth and metabolism, by acting as a sensor for nutrient and growth signals. mTOR functions through two complexes, mTOR complex 1 (mTORC1) and complex 2 (mTORC2), each impacting cell growth and survival differentially. Activation of mTORC1 is typically nutrient- and growth factor-dependent, mediated by signalling via the PI3K-Akt pathway, which in turn regulates cell growth by phosphorylating and inhibiting the tuberous sclerosis complex (TSC), a negative regulator of mTORC1.^44,48^ In *NF2*-SWN-related tumours, the loss of merlin leads to hyperactivation of mTORC1,^49,50^ contributing to tumour growth and progression. Similar mTORC1 dysregulation is a common abnormality observed in other cancers and solid tumours, often arising from mutations in PI3K/AKT signalling pathways, or loss of tumour suppressors such as PTEN.^51^ Mechanistically, merlin mediates mTORC1 inhibition by sequestering PIKE-L—an enhancer of PI3K-Akt signalling.^52^ Thus, the continued activity of mTORC1 in the absence of merlin promotes *CCDN1* (encoding Cyclin-D1) expression^53^—a crucial factor for cell cycle progression (Fig. 4). Targeting mTOR is a promising therapeutic approach for managing *NF2*-SWN-associated tumours but results, to date, have been inconsistent.^50,54,55^

### Hippo pathway

The Hippo pathway is a crucial regulator of organ development and tissue homeostasis, controlling cellular proliferation and survival through a kinase cascade involving MST1/2 and LATS1/2, which in turn regulate the activity of transcriptional coactivators YAP and TAZ.^56,57^ Activation of the Hippo pathway results in the phosphorylation and cytoplasmic retention of YAP and TAZ, preventing them from promoting the expression of genes associated with cell growth. In contrast, when the pathway is inactive, both YAP and TAZ enter the nucleus and activate genes that enhance cell proliferation and survival.^56,58,59^

**Figure 5:**
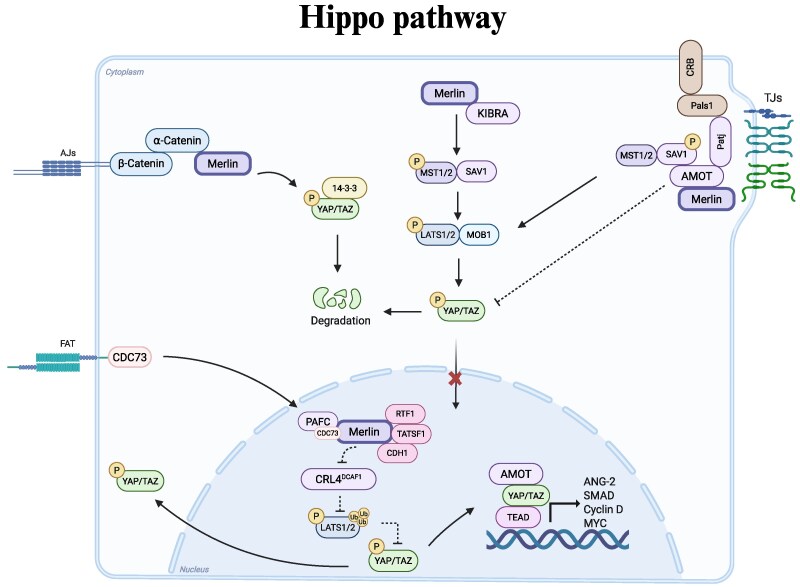
**Role of Merlin in Hippo signalling.** Merlin promotes Hippo pathway activation through interactions with adherens junctions, tight junctions and upstream scaffold proteins including KIBRA, AMOT and CRB–Pals1–Patj complexes. Activation of the MST1/2–SAV1 and LATS1/2–MOB1 kinase cascade leads to phosphorylation of YAP/TAZ, resulting in cytoplasmic retention through 14-3-3 binding, degradation and reduced transcriptional activity. When Merlin function is lost, YAP/TAZ can accumulate in the nucleus and interact with TEAD to drive expression of pro-growth genes, including ANG-2, SMAD, Cyclin D and MYC. Merlin also regulates nuclear tumour-suppressive mechanisms involving CRL4^DCAF1 and chromatin-associated transcriptional complexes. AJs, adherens junctions; AMOT, angiomotin; ANG-2, angiopoietin-2; β-catenin, beta-catenin; α-catenin, alpha-catenin; CDC73, cell division cycle 73; CDH1, cadherin 1; CRB, Crumbs cell polarity complex component; CRL4^DCAF1, cullin-RING E3 ubiquitin ligase 4–DDB1 and CUL4-associated factor 1; FAT, FAT atypical cadherin; KIBRA, kidney and brain expressed protein; LATS1/2, large tumour suppressor kinase 1/2; MOB1, MOB kinase activator 1; MST1/2, mammalian STE20-like protein kinase 1/2; MYC, MYC proto-oncogene; P, phosphorylation; PAFC, RNA polymerase II-associated factor complex; Pals1, protein associated with Lin seven 1; Patj, Pals1-associated tight junction protein; RTF1, RNA polymerase-associated protein RTF1; SAV1, salvador family WW domain-containing protein 1; SMAD, mothers against decapentaplegic homologues; TEAD, TEA domain transcription factor; TJs, tight junctions; Ub, ubiquitin; YAP/TAZ, yes-associated protein/transcriptional coactivator with PDZ-binding motif. Solid arrows indicate activation pathways, and dotted arrows/lines represent inhibitory effects. Created in BioRender. Done, R. (2026), https://BioRender.com/5x7kt78.

At the plasma membrane, merlin interacts with angiomotin to promote hippo pathway activity,^60^ by ensuring merlin remains in an open conformation.^61^ Beyond its role at the plasma membrane, within the nucleus, merlin blocks the activity of CRL4^DCAF1^ E3 ubiquitin ligases. Typically, this enzyme targets and polyubiquitinates LATS1/2 for proteasomal degradation.^62^ Pathogenic *NF2* mutants are unable to interact with CRL4^DCAF1^ leading to unchecked degradation of LATS1/2, allowing YAP/TAZ nuclear translocation and hyperproliferation (Fig. 5).^63^

Aberrant activation of the Hippo pathway is often seen in *NF2*-SWN patients, thus contributing to both tumorigenesis and immunoevasion. For example, overactivation of YAP/TAZ can upregulate *PTGS2* (encoding COX-2) expression,^64^ which is often overexpressed in *NF2*-SWN patients.^65^ Mechanistically, COX-2 works by metabolizing arachidonic acid into prostaglandin-E2, a powerful anti-inflammatory mediator, which can greatly impair dendritic cell maturation, cross-presentation,^66^ intratumoral CD8^+^ T-cell migration^67^ and increases the expression of immunoregulatory molecules.^68^ A phase 2 trial evaluating the efficacy of aspirin (a COX-1 and COX-2 inhibitor) to treat VS tumours is currently ongoing and is expected to complete in 2029.

## Histopathological features of vestibular schwannoma tumours

In 1920, Swedish neurologist Nils Ragnar Eugène Antoni first described the unique tissue architecture of schwannomas.^69,70^ Histological studies have since revealed consistent intratumoural heterogeneity, characterized by two distinct zones: Antoni A and Antoni B regions.

Antoni A regions are densely packed with spindle-shaped tumour cell nuclei arranged in a palisading pattern, alternating with acellular zones called verocay bodies.^71,72^ These regions also contain networks of cytoplasmic processes enclosed in a basement membrane rich in laminin and collagen IV—a hallmark of peripheral nerve sheath tumours.^73^ In contrast, Antoni B regions are loosely organized, are apparently less cellular and display features such as vascular changes (e.g. hyalinization, thrombosis and necrosis), often associated with degenerative processes known as ‘ancient change’.^74^

Between these zones lies a transitional region that represents the structural shift from Antoni A to Antoni B tissue. This transitional area is characterized by fragmented basal lamina and histological features reminiscent of Wallerian degeneration—a nerve injury response marked by axonal breakdown, myelin debris and macrophage-mediated clean-up.^75,76^ It also exhibits high cellular proliferation and is frequently enriched in macrophages, which actively phagocytose myelin and axonal debris, facilitating the restructuring of nerve fibres, consistent with active remodelling observed in Wallerian degeneration.^75,76^

## Vestibular schwannoma tumour-immune microenvironment

In addition to the indicated intertumoral heterogeneity, VS tumours display pronounced immune cell infiltration within the TME. Recognition of this immune landscape has informed recent stratification of VS into distinct molecular subtypes based on single-cell RNA sequencing (scRNA-seq) analyses.^77,78^ These studies have delineated ‘injury-like’ and ‘non-myelinating Schwann cell’ VS subtypes, as well as ‘neural crest’ and ‘immune enriched’ phenotypes but also revealed the broad diversity of immune populations within VS, including myeloid, lymphoid and dendritic cell lineages. scRNA-seq has further highlighted the heterogeneity of Schwann cell states within the tumours, such as myelinating, non-myelinating and stress-response Schwann cells.^77^ Furthermore, distinct macrophage subpopulations with variable activation states have also been identified, suggesting functional diversity within the immune compartment. Recent evidence suggests that immune activity within intracranial tumours may be influenced by the newly defined lymph node-skull-meninges-brain axis^18^ and, given the proximity of VS to ‘immune hubs’ of the meninges, could explain a route for immune cell trafficking between the tumour and the peripheral lymphoid tissues. Of particular interest, the ‘injury-like’ and ‘immune-enriched’ subtypes share a common feature: both represent immunologically ‘hot’ tumours, marked by substantial recruitment of myeloid and T-cells.^79,80^

Amongst the most abundant immune populations are tumour-associated macrophages (TAMs), which are consistently observed at high density within VS tissue and have been linked to tumour progression.^81^ Within this population, the alternatively activated (‘M2-like’) subset appears especially associated with rapid tumour growth, suggesting a potential pro-tumorigenic role.^82^ Ultimately, the ontogeny of TAMs in VS tumours is not fully understood. Whilst TAMs in many solid tumours arise from circulating monocytes, recent evidence points to additional contributions from tissue-resident populations. A PNS-resident macrophage subset with transcriptomic and developmental features of microglia (‘PNS microglia-like cells’) has been identified in large bodied vertebrates, including humans.^83^ This raises the possibility that TAMs in VS may comprise not only monocyte-derived cells but also PNS-resident macrophages with microglial-like characteristics.

Immunohistochemical^84,85^ and scRNA-seq analyses^77,78^ have confirmed that VS tumours harbour abundant CD4^+^ and CD8^+^ tumour-infiltrating lymphocyte (TILs). Spatial profiling from our laboratory has revealed that CD8^+^ T cells are often sequestered within TAM-rich perivascular niches in Antoni A regions, whereas in Antoni B regions, they are more frequently co-localized with pathogenic Schwann cell populations.^86^ Whilst these observations confirm that T-cells can effectively infiltrate VS tumours, relatively little is known about their functional identity, activation status or capacity to mediate anti-tumour responses. Some studies indicate that VS TILs exhibit molecular features consistent with reduced functional capacity^87^; however, the mechanisms underlying this remain poorly defined. Insights from other solid tumours suggest that factors such as the balance between effector and regulatory T-cell subsets, the local immunoregulatory milieu and the nature of the tumour antigenic landscape may critically shape T-cell protective anti-tumour responses. Understanding these variables within VS and in the context of *NF2*-SWN is essential for informing strategies to harness or restore T-cell function.

## T-cell responses against solid tumours—information relevant for *NF2*-SWN vestibular schwannoma

CD8^+^ T cells express and secrete cytolytic molecules such as perforin, granzymes and interferon gamma (IFNγ) and, as such, are key for eliminating cancer cells^88^ and form the backbone of cancer immunotherapy. Conversely, CD4^+^ T cells, whilst generally less cytotoxic than their CD8^+^ counterparts, have been shown to acquire effector functionality in certain conditions, particularly within the TME of solid tumours like melanoma.^89^ In addition to their potential for direct cytotoxicity, CD4^+^ T cells exhibit substantial functional plasticity, and thus, play a crucial role in sustaining effective anti-tumour immunity through various mechanisms. For example, CD4^+^ T cells ensure the efficient cross-presentation of tumour antigen by dendritic cells, to cytotoxic T-cells,^90^ and within the TME, help recruit cytotoxic CD8^+^ T-cells into the tumour in an IFNγ-dependent manner.^91^ Given the central role of CD8^+^ T-cells in directly eliminating tumour cells, CD8^+^ T-cells are the primary intended targets of modern immunotherapies. In particular, immune checkpoint blockade (ICB) against programmed death receptor-1 (PD-1), its ligand PD-L1 or cytotoxic lymphocyte antigen-4 (CTLA-4) act to reinvigorate exhausted CD8^+^ T-cells, restoring their cytolytic capacity within the TME.^92,93^ This therapeutic focus on CD8^+^ T-cells has yielded durable clinical benefit across multiple solid tumours, including melanoma and lung cancer.^94^

**Figure 6:**
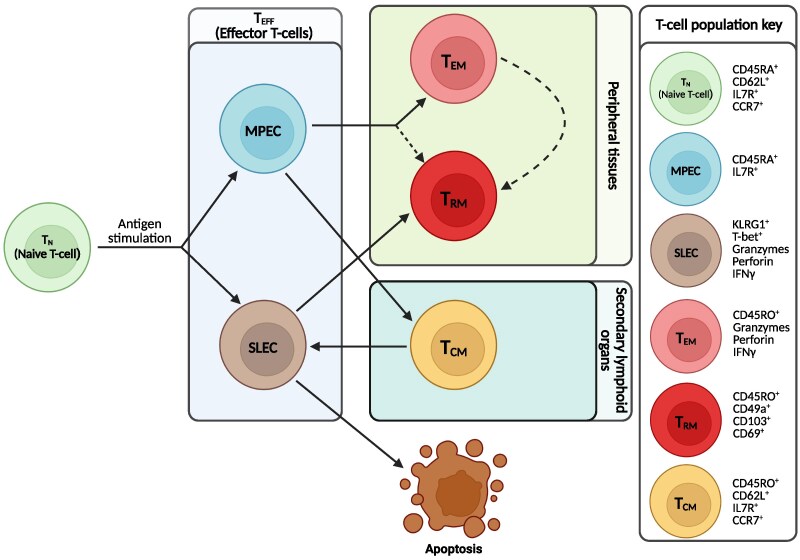
**Pathways of T-cell differentiation into effector and memory subsets.** Naïve T cells (T_N_; CD45RA^+^CD62L^+^CCR7^+^IL7R^+^) are primed in secondary lymphoid organs, activate and expand into Effector T cells (T_EFF_). Some T_EFF_s lose the expression of CD62L, IL7R, CCR7 and gain the expression of T-bet and KLRG1 to become SLECs. This population dominates the acute response and exhibits heightened cytolytic capability but are also subject to apoptosis within the contraction phase. Conversely, some T_EFF_s retain the expression of IL7R, becoming memory precursor effector cells (MPECs). MPECs seed long-lived memory lineages. From MPECs, central memory T cells (T_CM_; CD45RO^+^CD62L^+^CCR7^+^IL7R^+^) circulate between lymphoid organs and the blood, with high proliferative recall. Effector memory T cells (T_EM_; CD45RO^+^CD62L^−^CCR7^−^) circulate between the blood and inflamed tissues, poised for rapid effector function, expressing cytolytic molecules. Tissue-resident memory T cells (T_RM_; CD45RO^+^CD69^+^CD103^+^CD49a^+^) arise from KLRG1-expressing effectors within inflamed tissues under local cytokine milieu (TGFβ, IL-15) and persist long term within tissues. Solid lines denote dominant differentiation routes, whilst dashed arrows indicate plasticity during recall. IL7R, interleukin-7 receptor; KLRG1, killer cell lectin-like receptor subfamily G member 1; T-bet, T-box transcription factor TBX21; IFNγ, interferon gamma; TGFβ, transforming growth factor beta. Created in BioRender. Done, R. (2026), https://BioRender.com/u1g4h6l.

Following activation, naïve T (T_N_) cells, undergo three phases: (i) clonal expansion and gain of effector function, (ii) a contraction phase and (iii) maturation into, and maintenance of memory populations, which persist after the initial immune response and provide a rapid and robust responses upon neoantigen re-exposure,^95,96^ as depicted in Fig. 6. T_N_ cells are characterized by the expression of CD62L, CD127 (IL-7R) and CCR7, which provide T_N_ cells with the ability to circulate through the lymphatic system. Following antigenic challenge, T_N_ cells activate into effector T (T_EFF_) cells. However, this process is not strictly linear and is shaped by a combination of deterministic and stochastic signals. Some T_EFF_ cells differentiate into short-lived effector cells (SLECs) characterized by expression of the marker KLRG1. Controlling this lineage fate trajectory is the transcription factor T-box expressed in T cells (T-bet), induced by IL-12.^97^ SLECs represent the ‘peak’ activation state of the CD8^+^ effector lineage and are cytotoxic, yet they are also the population mostly affected by the contraction phase. Conversely, following activation a small subset of T_EFF_ cells retains IL-7R expression, thus predisposing them to become memory-precursor effector cells (MPECs),^98^ which is a pivotal step in forming both central memory (T_CM_) and effector memory (T_EM_) T-cells.^99^ T_CM_ and T_EM_ populations differ by phenotype and the expression of homing markers CD62L and CCR7^100-102^: T_CM_ cells maintain CD62L and CCR7 expression, enabling their circulation between blood and secondary lymphoid organs, whereas T_EM_ cells downregulate CD62L and CCR7 and their circulation is largely restricted to the blood and inflamed tissues.^103^ Functionally, T_CM_ cells are epigenetically primed to rapidly produce IL-2 and IL-12 and possess high proliferative capacity upon antigenic rechallenge.^102^ Conversely, T_EM_ cells exhibit low proliferative capacity but excel in gaining rapid effector function, by producing IFNγ and cytolytic molecules like perforin and granzyme B upon antigen re-encounter. Whilst cytotoxic CD8^+^ T-cells are essential for mediating anti-tumour-immune responses, their ability to sustain long-term tumour control and tumour immunosurveillance relies heavily on the presence of memory T-cells.

The comparative impact of T_CM_ and T_EM_ populations on tumour immunity reflects these distinct functional specializations. T_CM_ cells, with their ability to home to secondary lymphoid organs, receive sustained survival and co-stimulatory signals which support long-term persistence and rapid expansion upon antigen re-encounter. This property makes them particularly effective at maintaining durable tumour control and has been linked to enhanced responsiveness to ICB with anti-PD-1.^104-106^ In contrast, T_EM_ cells which predominantly reside within peripheral tissues, including tumours, can deliver immediate effector responses at the site of disease through rapid cytokine production and direct cytolysis. Such on-site activity likely underlies observations that intratumoural CD8^+^ T_EM_ populations also correlate with improved checkpoint blockade efficacy compared to their counterparts in tumour-draining lymph nodes.^107^ Whilst T_CM_ appear more critical for sustaining long-term immunity, and T_EM_ for delivering rapid cytotoxic pressure, optimal ICBoutcomes are observed when both subsets are present—T_CM_ ensuring proliferative replenishment and T_EM_ exerting immediate tumour control. Consistently, the combined abundance of CD45RO-expressing T_CM_ and T_EM_ cells has been associated with heightened ICB sensitivity and improved survival.^108^

Recently, tissue-resident memory (T_RM_) cells have also emerged as integral components of anti-tumour immunity. These cells, characterized by their expression of CD49a, CD69 and CD103, are primarily located in peripheral tissues where they provide rapid local immune defence.^109^ The expression of CD103 promotes T_RM_ adhesion to epithelial cells via the binding of E-cadherin,^110,111^ whilst CD49a support their retention in type IV collagen-rich extracellular matrices, enhancing stability within the TME.^112^ CD69 further enforces tissue residency by suppressing S1PR1 expression, thereby preventing egress from peripheral tissues.^110^ Beyond their unique phenotype, T_RM_ cells are also distinguished from other memory subsets by their unique developmental pathways. Like T_CM_ and T_EM_ subsets, T_RM_ cells also develop from T_N_ cells, but transition through a unique early effector CD8^+^ subset, characterized as KLRG1^+^ precursors,^110^ similar to that of T_CM_ cells. Once developing, T_RM_ cells seed the peripheral tissues and, supported by local signals such as TGFβ and IL-15, upregulate CD103 to drive their retention and adaptation.^110,111^ Unlike T_CM_ and T_EM_ populations, T_RM_ cells do not re-circulate, instead they persist long term at barrier and peripheral sites, where they provide rapid, localized protective capacity completely independent of continual replenishment from memory circulating pools.^109,111^

In the context of tumour control, T_RM_ cells are recognized for their potent cytotoxic activity. Elevated intratumoural T_RM_ frequencies are consistently associated with favourable clinical outcomes in both lung cancer and melanoma.^113,114^ Functionally, T_RM_ exhibit a hybrid profile that integrates features of both T_CM_ and T_EM_ populations. Like T_CM_ cells, T_RM_ retain the capacity to proliferate *in situ* following antigenic re-encounter, thereby supporting the local expansion of tumour neoantigen-specific T cells within the TME.^115^ At the same time, they parallel T_EM_ in being transcriptionally poised for rapid effector activity, as demonstrated by CD49a^+^ T_RM_ in human skin, which rapidly deploy perforin and granzyme B upon stimulation.^116^ This unique combination of proliferative potential and immediate cytotoxic readiness equips T_RM_ with a dual advantage in anti-tumour immunity. Notably, ICB, with anti-PD-1, has been shown to preferentially expand and activate T_RM_ populations, further enhancing tumour regression and cytolysis in ovalbumin-expressing melanoma and colorectal tumour models.^117^

Whilst the presence and functional relevance of T_CM_, T_EM_ and T_RM_ populations in *NF2*-SWN-associated VS tumours remains to be addressed, the critical roles of the T-cell populations in other tumour types suggest the potential impact of targeting memory subsets to enhance anti-tumour responses against VS tumours.

## T-cell exhaustion

**Figure 7:**
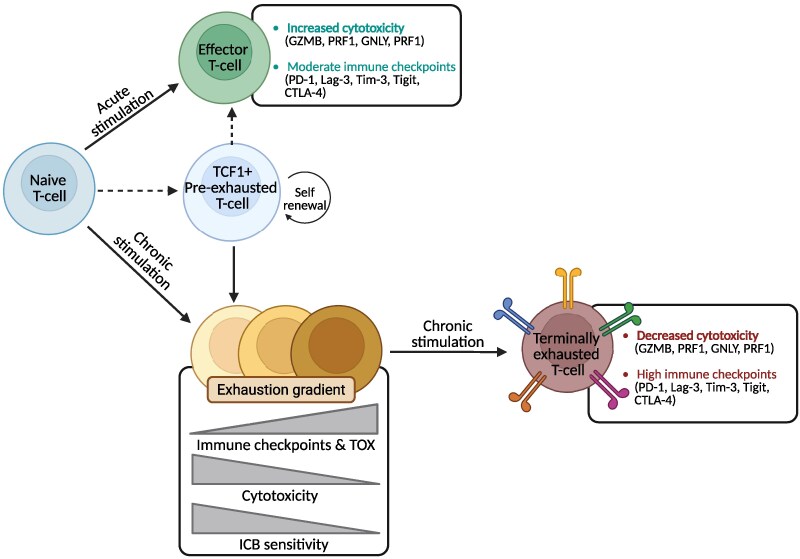
**Differentiation and functional spectrum of exhausted T cells.** Naïve T cells differentiate into effector cells under acute antigen stimulation, acquiring high cytotoxic capacity (granzymes, perforin and granulysin) with moderate expression of immune checkpoint receptors (PD-1, LAG-3, TIM-3, TIGIT, CTLA-4). Under chronic stimulation, a progenitor exhausted (T_PEX_; TCF1^+^) population emerges that retains proliferative capacity and self-renewal, whilst sustaining both effector and exhausted populations. These cells give rise to terminally exhausted (T_EX_; TOX^+^) cells, which exhibit markedly reduced cytotoxicity, high expression of immune checkpoint receptors, and diminished responsiveness to immune checkpoint blockade. The process reflects a continuum of differentiation, ranging from stem-like TCF1-expressing progenitors to terminally dysfunctional T_EX_ subsets. Dashed arrow represents an indirect pathway and solid arrows illustrate a direct pathway. GZMB, granzyme B; PRF1, perforin 1; GNLY, granulysin; TCF1, T-cell factor 1; TOX, thymocyte selection-associated high mobility group box protein; PD-1, programmed cell death protein 1; LAG-3, lymphocyte activation gene 3; TIM-3, T-cell immunoglobulin and mucin domain-containing protein 3; TIGIT, T-cell immunoreceptor with Ig and ITIM domains; CTLA-4, cytotoxic T-lymphocyte-associated protein 4; ICB, immune checkpoint blockade. Created in BioRender. Done, R. (2026), https://BioRender.com/a1tzw98.

One of the major barriers to effective T-cell function within the TME is T-cell exhaustion. First described in chronic viral infection^118^ and subsequently extended to tumours,^119^ exhaustion is a progressive T-cell dysfunction programme driven by chronic antigenic stimulation and sustained inhibitory receptor signalling.^120^ Exhausted T-cells are characterized by reduced proliferative capacity, diminished effector function and persistent co-expression of inhibitory checkpoint receptors (described in more detail below and in Fig. 7).

Exhaustion is now recognized as a spectrum ranging from precursor exhausted (T_PEX_) cells to terminally exhausted (T_EX_) cells. T_PEX_ cells, defined by expression of the stem-associated transcription factor TCF1 (encoded by *Tcf7*), retain the ability to self-renew and can act as a reservoir of tumour-specific T-cells.^121,122^ They are also capable of trafficking between tumour-draining lymph nodes (tdLNs) and tumour sites, enabling the replenishment of the intratumoural pool with tumour-specific effector T-cells.^123^ In contrast, T_EX_ cells represent the endpoint of the exhaustion programme. Their identity is enforced by the transcription factor thymocyte selection-associated high mobility group box protein (TOX), which drives epigenetic remodelling, priming exhaustion-associated genes,^124^ and prevents the degradation of immune checkpoint receptors.^125^ T_EX_ cells are further characterized by their severely reduced functionality: they lack proliferative potential, secrete minimal IL-2 and display impaired cytotoxicity. Consequently, T_EX_ cells are poorly responsive to ICB and incapable of sustaining durable anti-tumour immunity.^126^ During chronic antigenic exposure, a PD-1^+^TCF1^+^ ‘stem-like’ progenitor T_PEX_ population arises early at priming in tdLNs‚ and does not derive from established memory cells, although they resemble T_CM_ subsets.^127^ Fate-mapping and adoptive-transfer studies show that this progenitor branches from the primed pool of T-cells, and differentiates into T_EFF_-like and T_EX_ cells,^128,129^ whereas classical memory differentiation is favoured following antigen clearance.

Immune checkpoint receptor signalling represents one of the most prominent mechanisms of CD8^+^ T-cell immunosuppression within the TME. These pathways normally serve to fine-tune T-cell responses and prevent immunopathology during inflammatory contexts.^130^ In tumours, persistent antigenic exposure drives sustained immune checkpoint receptor upregulation on CD8^+^ T-cells, which are engaged by ligands presented by various cell populations within the TME, which directly enforces the exhausted T_EX_ phenotype, ‘switching off’ anti-tumour T-cells. PD-1 is a particularly well characterized immune checkpoint receptor in this respect. By attenuating both CD28 co-stimulatory signals^131^ and T-cell receptor signalling cascades,^132^ PD-1 suppresses effector function and locks T_EX_ cells into a dysfunctional state. Other inhibitory receptors such as the ‘classical’ (PD-1 and CTLA-4) and ‘alternative’ (LAG-3, TIGIT and TIM-3) molecules all act through similar mechanisms to restrict T-cell activation. Importantly, immune checkpoint expression is not exclusive to T_EX_ cells but is found on all antigen-experienced T-cells. Thus, the co-expression of multiple checkpoint receptors, rather than any single receptor alone, is a defining feature of T-cell exhaustion.^133,134^ This also explains why blockade of only one pathway, such as PD-1, may be insufficient to fully restore T-cell function within tumours and is the reason why identification of optimal combinatorial treatments is currently dominating immune-oncology clinical trials.

The discovery of PD-1 by Honjo and colleagues^135,136^ provided the foundation for a therapeutic strategy that has since transformed immune-oncology. Antibodies blocking PD-1 or CTLA-4 have delivered durable clinical responses across multiple malignancies including melanoma, colorectal and gastric cancer.^137,138^ These successes demonstrated the therapeutic potential of reversing checkpoint-mediated dysfunction. However, as previously discussed, not all exhausted T-cells are equally responsive to ICB. Clinical efficacy is increasingly recognized to depend on the presence of TCF1^+^ T_PEX_ cells, which do retain proliferative capacity and are the critical population mediating therapeutic benefit.^130^ Following PD-1 blockade, T_PEX_ cells undergo a proliferative burst, generating large numbers of reinvigorated effectors that repopulate the TME.^139^ By contrast, T_EX_ cells, driven by TOX, remain functionally fixed and unresponsive to ICB. Connolly *et al*. illustrate that these stem-like CD8^+^ T cells persist within tdLNs, serving as the primary source of anti-tumour T-cells.^122^. Furthermore, recent comprehensive reviews^140^illustrate that failure to sustain such proliferative T_PEX_ cells leads to a collapse of anti-tumour T-cell immunity, whilst Lei *et al*. highlight how tdLNs serve as the critical niche for housing T_PEX_ cells that underpin effective immunotherapy responses.^141^

Although T_PEX_ and T_EX_ populations have not yet been defined in *NF2*-SWN-associated VS, existing evidence from spatial profiling illustrates that CD8^+^ TILs often co-express PD-1 and TIM-3 in VS,^86^ suggesting the emergence of a partially exhaustive phenotype, following infiltration into the tumour. It is therefore plausible that VS-specific T-cells undergo a similar trajectory towards exhaustion, with functional outcomes shaped by checkpoint ligand expression within the tumour. Identifying whether VS TILs include TCF1^+^ stem-like populations—either within the tumour or tdLNs—would provide valuable insight into their therapeutic potential and responsiveness to ICB.

## Immunosuppressive features of *NF2*-SWN-associated vestibular schwannoma tumours

Whilst *NF2*-SWN-associated VS TILs may follow an exhaustion trajectory under chronic antigenic stimulation, the extent of their dysfunction is dictated by their contexture and the signals they receive within the TME. Inhibitory receptors such as PD-1 and TIM-3 are commonly expressed on antigen-experienced T-cells; however, it is their sustained engagement with tumour- or stroma-derived ligands that drives durable T-cell functional impairment. VS tumours exemplify this, providing an immunosuppressive landscape in which multiple mechanisms—including checkpoint receptor-ligand interactions, regulatory T cells, immunomodulatory cytokines and metabolic constraints—converge to shape the trajectory and efficacy of VS-specific T-cell responses.

### Immune checkpoint receptor-ligand interactions

The PD-1/PD-L1 axis appears to be a dominant suppressive pathway in *NF2*-SWN tumours. PD-L1 is strongly expressed in *NF2*-SWN-related VS^142^ and meningiomas,^143^ and in VS, its expression correlates with CD8^+^ T-cell infiltration.^142^ PD-L1^+^ Schwann cells co-localize with intratumoural CD8^+^ T-cells,^86^ consistent with direct Schwann cell engagement of T-cells. Beyond tumour cells, TAMs are a plausible additional PD-L1 source. In other solid tumours, PD-L1 frequently localizes to macrophages, and macrophage-restricted PD-L1 can suppress CD8^+^ T-cell function; conditional loss of macrophage PD-L1 restores anti-tumour CD8^+^ responses.^144^ In melanoma models, PD-L1^+^ TAMs engage PD-1 on CD8^+^ TILs^145^ and form prolonged, weak, antigen-dependent contact which license T-cell exhaustion.^146^ Mechanistically, PD-L1 upregulation in *NF2*-SWN may be linked to dysregulated PI3K-AKT-mTOR and Hippo signalling.^143,147^ Although translational evidence is preliminary: a case report notes VS growth arrest with anti-PD-1 monotherapy,^148^ and in a CPA mouse model, combined PD-1 and vascular endothelial growth factor (VEGF) blockade outperformed PD-1 or VEGF monotherapy alone.^149^ Together, these data support PD-1/PD-L1 as a tractable immunosuppressive pathway and a rational therapeutic target in *NF2*-SWN.

Whilst PD-1 and PD-L1 remain the most intensively studied, other checkpoint-ligand interactions are also implicated in shaping T-cell dysfunction. TIM-3, for example, is highly expressed on exhausted T-cells, but its ligands are unusually heterogeneous, including galectin-9, CEACAM1, phosphatidylserine and HMGB1, each of which can independently suppress T-cell responses.^150^ Furthermore, LAG-3 competes with CD4 for binding to MHC class II molecules, acting as a negative regulator of T-cell activation,^151^ whilst TIGIT engages poliovirus receptor (PVR, also known as CD155) and nectin family ligands,^152^ leading to the suppression of T-cell activity. In malignant schwannomas, PD-1 expression alone was not sufficient to identify exhausted TILs; rather, co-expression with TIM-3 was more predictive of dysfunction.^153^ Taken together, the PD-1/PD-L1 axis emerges as the most probable ligand-driven suppressive pathway in VS tumours. However, whether additional checkpoint-ligand interactions such as TIM-3, LAG-3, CTLA-4 or TIGIT also contribute to T-cell dysfunction in these tumours remains unresolved. As current evidence remains limited to a narrow set of T-cell exhaustion markers, a more comprehensive mapping of checkpoint receptor-ligand networks will be critical both to delineate the immunoregulatory landscape of VS and to inform the rational design of combination immunotherapies capable of reversing T-cell exhaustion.

### Foxp3^+^ T regulatory cells

In addition to effector and exhausted CD8^+^ T cells, canonical *FoxP3^+^* Treg populations have also been identified within VS tumours via both scRNA-seq^77,78^ and immunohistochemical analyses of CD4 and CD25 (IL2RA).^84^ The immunosuppressive role of FoxP3^+^ Tregs in the TME is well documented,^154^ with their presence associated with impaired anti-tumour T-cell responses. For instance, a meta-analyses across 76 studies utilizing 17 different cancer types confirmed that elevated intratumoural *FoxP3^+^* Tregs correlate with worse overall survival,^155^ and their depletion has been shown to improve T_EFF_ function and reduce exhaustion signatures.^156^

*FoxP3^+^* Tregs within VS tumours transcriptionally upregulate CTLA-4, a feature that reinforces their suppressive capacity in both *in vitro* and *in vivo* settings.^157^ This has important therapeutic implications, as immune checkpoint blockade may exert dual effects in the VS TME; not only reinvigorating exhausted cytotoxic T-cells but also destabilizing Treg function by impairing their lineage stability or reprogramming them into inflammatory phenotypes. Such ‘double-hit’ effects of ICB have been observed in other solid tumours and may be critical for overcoming the immunosuppressive niche characteristic of *NF2*-SWN-associated VS.^158^

### Hypoxia and metabolic constraints

Although direct evidence in VS remains limited, findings from other solid tumour types suggest that hypoxia and metabolic stress are key drivers of T-cell dysfunction, and similar mechanisms may plausibly operate within the VS TME. In particular, hypoxia has been shown to synergize with nutrient deprivation to promote the expression of immune checkpoints such as PD-1, CTLA-4 and TIM-3, leading to the development of an exhausted T-cell phenotype and reduced anti-tumour immunity.^159^ In VS specifically, the presence of hypoxic regions is supported by increased expression of HIF-1α in more than half (55%) of *NF2*-SWN-associated schwannomas.^85^ These hypoxic zones may impair T-cell responses both directly—by promoting mitochondrial dysfunction^160^—and indirectly through the activity of TAMs, which contribute to HIF-1a upregulation and metabolically suppress T-cell proliferation by depleting extracellular L-arginine, a nutrient essential for T-cell functionality.^161^

Beyond hypoxia, tumours can further enforce metabolic constraints on TILs through altered metabolism. Whilst this phenomenon has not yet been confirmed in VS, many solid tumours preferentially utilize anaerobic glycolysis, even in the presence of oxygen (Warburg effect). This allows tumours to rapidly take up glucose and produce toxic oncometabolites such as lactate, which acidifies the TME.^162^ This acidic environment has been shown to impair T-cell motility, cytotoxicity^163,164^ and decrease responsiveness to immune checkpoint blockade^165^ in other tumour models. Though similar glycolytic reprogramming and acidification in VS remain speculative, the presence of TILs, hypoxic signalling and gene clusters,^77^ alongside immunosuppressive TAM activity raises the possibility that T-cells in VS may face comparable metabolic barriers. Notably, imaging mass cytometry with integrated scRNA-seq in *NF2*-SWN VS shows that intratumoural T-cell populations (both CD8^+^ and CD4^+^) express the lactate transporter NCT4/SLC16A3, with lower GLUT1/MCT4 on Schwann cells, consistent with TIL adaptation to a lactate-rich niche.^86^

### Cytokines and chemokines

Cytokines act as critical chemical mediators to orchestrate immune activation or regulation. Thus, a comprehensive understanding of the cytokine milieu in VS tumours is critical for advancing immunotherapeutic strategies and enhancing anti-tumour T-cell responses. Evidence, to date, suggests that VS tumours exhibit a mixed cytokine signature, containing both pro (TNF, IL-1β and IL-6) and anti-inflammatory (TGFβ) mediators.^166,167^ In a small immunohistochemical study, Taurone *et al.* reported an increased immunoreactivity for TGFβ which is largely restricted to Antoni A regions.^166^ Similarly, IL-1b, IL-6 and VEGF expression was greater in VS tissue when compared to healthy nerve; however, the expression of these cytokines was restricted to the Schwann cell cytoplasm and did not exhibit any major differences in spatial distribution across the different histomorphic regions.^166^

The elevated expression of TGFβ in VS tumours is likely to play a pathogenic role in tumour pathogenesis and progression. Both TGFβ1 and TGFβ2 isoforms have been identified as potent mitogens for Schwann cells, capable of promoting cellular proliferation through activation of latent TGFβ complexes.^168^ This mitogenic response appears to be largely mediated via TGFβRII, as genetic ablation of TGFβRII renders Schwann cells more susceptible to apoptosis whilst simultaneously attenuating their proliferative capacity,^169^ highlighting the centrality of this receptor in TGFβ-dependent cellular dynamics during nerve development, and potentially during tumorigenesis. Beyond its role in Schwann cell biology, TGFβ exerts pleiotropic effects in various tumour contexts and is broadly considered to have pro-tumorigenic properties. It has been implicated in promoting tumour cell migration, invasion and dissemination, establishing itself as a critical driver of tumour progression across cancers.^170^ Importantly, TGFβ also shapes the TME, exerting profound immunosuppressive effects, particularly on T-cell-mediated anti-tumour responses. TGFβ suppresses the cytotoxic programme of CD8^+^ T-cells, impairing effector function.^171^ Furthermore, TGFβ signalling enhances PD-1 expression, thereby fostering T-cell exhaustion.^172^ This immunoregulatory axis has therapeutic implications. Notably, dual blockade of TGFβ and PD-1 has demonstrated synergistic efficacy in preclinical mammary carcinoma models, restoring anti-tumour immunity by expanding a pool of stem-like CD8^+^ T-cells with enhanced cytotoxic potential whilst reducing the prevalence of T_PEX_ cells.^173^ Whilst the effect of intratumoural TGFβ signalling on T-cells in VS has not been interrogated, these findings suggest that targeting TGFβ signalling, particularly in immunologically ‘hot’ tumours, may represent a promising avenue to enhance T-cell-mediated responses.

Alongside the increased presence of TGFβ in the VS TME, immunohistochemical analyses also reveal significantly elevated levels of IL-6 in VS tissue compared to normal nerve.^166^ This is notable given IL-6’s role in promoting pro-inflammatory gene signatures in Schwann cells via AP-1 activation.^174^ Such signalling is associated with Schwann cell differentiation and demyelination, a process that may underline the morphological distinctions observed between Antoni A and B regions of VS tumours—features reminiscent of Wallerian degeneration. Interestingly, blocking IL-6 is associated with control of both tumour burden and nociception in a patient-derived xenograft model of VS.^172^

Beyond its neurobiological effects, IL-6 also plays a multifaceted role in shaping T-cell responses within the tumour context. Macrophage-derived IL-6 has been shown to drive T-cell exhaustion through STAT3-dependent upregulation of immune checkpoints such as PD-1.^175^ Supporting this, co-blockade of IL-6 and PD-1 in hepatocellular carcinoma models has led to improved tumour control, whilst IL-6 deletion in hepatocellular carcinoma and lung cancer-bearing mice prevents the onset of T-cell exhaustion and enhances effector CD8^+^ T-cell responses.^176,177^ Together, these findings suggest that IL-6 may play a dual role in shaping the VS TME—by influencing Schwann cells, contributing to tumour-associated nerve degeneration, and by driving immunosuppressive signalling which drives T-cell exhaustion. As such, targeting of IL-6 signalling may represent a promising avenue to both modulate the tumour stroma and reinvigorate anti-tumour T-cell responses.

TNFα is also another pro-inflammatory mediator which exhibits pleiotropic roles within VS. VS tumours exhibit heightened expression of TNFa when compared to normal nerves.^166,178^ Concomitant with these results, Diwali *et al*. also show a heightened expression of TNFRII—one of two putative receptors for TNFα—within the VS TME.^179^ Interestingly, TNFα is thought to induce NFκB signalling via TNFRII on Schwann cells which drives their proliferation, and inhibition of NFκB signalling significantly perturbs the growth of primary VS cell cultures *in vitro*. Furthermore, one significant symptom associated with the growth of VS tumours is the loss of hearing, VS-derived TNFα has been liked to direct ototoxicity via cochlear hair cell death, and neutralization of TNFα has been shown to improve hair cell survival in cochlear explant models.^180^ Illustrating the potential negative role TNFα has on the development of VS tumours and hearing loss *in vivo*.

Whilst TNFα may contribute to VS tumorigenesis and hearing loss, chronic TNFα signalling also suppresses anti-tumour immunity. In a murine melanoma model, TNFRI-dependent TNFα signalling in CD8^+^ T-cells prevented their intratumoural accumulation. Blockade of TNFRI not only enhanced CD8^+^ T-cell infiltration but also synergized with anti-PD-1 therapy to prevent upregulation of exhaustion markers such as TIM-3 and PD-L1, thereby improving tumour control and survival.^181^ Beyond this, TNFα can also directly induce PD-L1 expression in both malignant cells^182,183^ and macrophages,^184^ further reinforcing immunosuppression. Collectively, these findings highlight TNFα as a central regulator of both VS tumour cell biology and the immune landscape, linking tumour growth, hearing loss and impaired T-cell function.

Beyond the role of TNFα, there is growing evidence to suggest that the IL-1/IL-1β axis is active in human VS and contributes to shaping the immune TME. Immunohistochemical assays have demonstrated an increased positive stain for IL-1β in VS compared to normal nerve.^166^ Complementing these findings, scRNA-seq has identified a subset of IL-1β-producing TAMs within VS, with the expression of IL-1β correlating positively with tumour growth.^185,186^

Insights from other tumour models further highlight the immunosuppressive consequences of IL-1β signalling. In ovarian cancer, TAMs release IL-1β that drives PD-L1 upregulation on tumour cells, establishing a suppressive feedback loop limiting T-cell mediated immunity.^187^ Furthermore, preclinical studies illustrate that blockade of IL-1β enhances a-PD-1 efficacy, resulting in increased intertumoral expression of granzyme B, restoring cytotoxic T-cell function.^187^ Similarly, in murine breast cancer models, IL-1β expression promoted macrophage infiltration via CCL2-dependent chemotaxis, whereas dual blockade of PD-1 and IL-1β synergistically increased CD8^+^ T-cell infiltration and suppressed tumour growth.^188^ Together, these findings suggest that IL-1β signalling is not only present, but functionally relevant in VS. By fostering a TAM-dominated immunosuppressive niche, IL-1β may limit effective anti-tumour T-cell responses. Thus, therapeutic co-targeting of the IL-1/IL-1β axis in combination with ICB represents a rational strategy to enhance T-cell mediated anti-tumour immunity.

In summary, the cytokine landscape of VS tumours is complex and consequential, shaping tumour progression, stromal remodelling and anti-tumour-immune responses. Notably, recent evidence indicates that the cytokine and chemokine dysregulation in VS is not confined to the tumour bed. A targeted proteomic analysis of patient plasma demonstrates that individuals with growing VS exhibit elevated circulating chemokines (CCL2, CXCL9 and CXCL10),^189^ suggesting that cytokine signalling in VS extends systemically and may facilitate ongoing immune recruitment or chronic inflammation. Thus, these findings suggest that the cytokine milieu in VS is not a by-product of tumour growth, rather an active participant in immunoevasion. Therefore, therapeutic strategies that target cytokine signalling, in combination with immune checkpoint blockade, may offer a promising therapeutic avenue to slow tumour progression and boost anti-tumour T-cell responses.

## Preclinical animal models to investigate *NF2*-SWN

The study of *NF2*-SWN requires an integrative, multidisciplinary approach that leverages both human clinical samples and preclinical animal models. Human tumour samples provide essential insight into the molecular and histopathological characteristics of *NF2*-SWN-associated VS but offer limited information regarding tumour pathogenesis, drug responses and mechanistic validation of tumour-host interactions. To address these limitations, several preclinical animal models spanning genetically engineered murine models, syngeneic implantation models and xenograft models have been developed, each with unique advantages and challenges, summarized in Table 3.

**Table 3:** Summary of the available preclinical animal models used to study NF2-associated VS tumours

Model	Advantages	Disadvantages
Human tumour samples	Provides translational insights into molecular and histopathological characteristics of *NF2*-associated VS	Lacks information on tumour pathogenesis, drug response and tumour-immune interactions
Postn-Cre; *Nf2^flox/flox^* Murine model.^185^	Provide an accurate representation of human NF2 VS growth and pathologies	Prolonged latency, asynchronous tumour growth with high financial costs—making this a difficult model for high throughput NF2 research
Human-derived HEI-193 immortalized schwannoma cell lines^190^	High translational relevance with imaging-compatible luciferase reporters allows for non-invasive monitoring	Peripheral tumour localization with no intracranial TME Mice are immunodeficient so unable to study tumour-immune interactions
SC4-9Luc Schwann cell allograft model (xenograft)^191^	Anatomical accuracy of tumour implantation Compatible with imaging modalities Allow for functional testing such as hearing preservation	Short experimental timeline (∼25 days) Extended recovery time following tumour implantation Mice are immunodeficient
CPA injection models (syngeneic)^186,187^	Anatomical accuracy of tumour, histological accuracy, comparable with imaging modalities Can be used to study the tumour-immune interactions (Chen *et al.* model only)	Immunodeficient model thus cannot study the immune interaction (Dinh *et al.* model only)

The Postn-Cre; *Nf2*^flox/flox^, genetically engineered murine model developed by Gehlhausen *et al.*,^190^ closely resembles *NF2*-SWN pathology, by virtue of *Nf2* exon 2 deletion, with VS tumours developing after ∼6 months. These tumours display histopathological similarities to human VS, including Antoni A and Antoni B regions. However, the long latency and asynchronous tumour growth in this model pose logistical and financial challenges for large-scale experimental studies.

Dinh *et al.*^191^ and Chen *et al*.^192^ developed murine orthotopic syngeneic implantation tumour models that involve the implantation of merlin-deficient Schwann cells into the CPA, closely replicating the biology of human VS. These models derive from conditional excision of *Nf2* exon-2, which is the most widely used genetic strategy to model merlin loss in Schwann cells, and drives schwannoma formation. Furthermore, the mutant Schwann cells employed by Chen *et al*. also contain cleavable luciferase, allowing to measure luciferase signal in the blood, which is directly proportional to the size of the tumour.^192^ Importantly, the CPA model optimized by Chen *et al*. utilizes immunocompetent (mixed C57BL/6×FVB) mice, allowing for focused study of tumour-immune dynamics and testing of T-cell-based immunotherapies, making this an attractive choice for exploring immunotherapeutic strategies. Some studies utilizing this model evaluating drugs such as losartan,^193^ VEGF blockade^149^ and impact of irradiation^194^ in combination with anti-PD-1 immunotherapy have revealed favourable responses in both tumour growth and T-cell infiltration.

In contrast, xenograft VS models developed by Saydam *et al*.^195^ involve transplantation of human-derived schwannoma cells onto the mouse sciatic nerve. These models commonly incorporate Gaussia luciferase (GLuc) reporters, enabling non-invasive, bioluminescent tracking of tumour growth and response to therapy. Whilst highly translationally relevant and amenable to imaging, these models have limitations: the peripheral tumour location does not recapitulate the intracranial VS environment, and the use of immunodeficient hosts precludes investigation of tumour-immune dynamics.

The SC4-9luc Schwann cell allograft model, as detailed by Bonne *et al.*,^196^ accurately replicates the site of human VS, as mutant *Nf2* Schwann cells are implanted into the CPA region of mice and allows for real-time tumour trafficking and auditory function assessment. These Schwann cells are spontaneously transformed Schwann cells isolated from adult *Nf2*^KO3/flox2^ mouse sciatic nerve. However, the model’s short experimental timeline and the implantation into immunodeficient NU/NU mice limit its use for studying tumour-immune interactions over time.

## Current and emerging treatment strategies for *NF2*-SWN-associated vestibular schwannoma

Building upon insights from both clinical trials and preclinical models, we summarize the current and emerging therapeutic strategies for the treatment of VS. To date, most relevant interventions have focused on angiogenesis or tumour intrinsic signalling rather than directly modulating anti-tumour immunity. Nevertheless, many of these treatments will likely exert immunological effects given their ability to reshape the TME. Supplementary Table 1 summarizes clinical and translational studies in VS, including ICB, anti-angiogenic therapies and targeted agents. Collectively, these studies provide an initial framework for rational immunotherapy development and highlight opportunities for combination strategies.

Notably, when compared with many other solid tumours, few immunotherapy approaches have been attempted in VS, with a small subset of immune-directed interventions having been tested in preclinical models.^194^ This highlights the need for focussed and detailed research in this area and prioritizing functional analyses of the VS TME will be critical for identifying suppressive interactions that are therapeutically tractable.

## Conclusion and future directions

Recent studies have begun to shed light on the immune environment within VS tumours, classifying many VS as immunologically hot tumours, due to their noticeable infiltration of macrophages and T-cells. However, despite the presence of these immune populations, the effectiveness of T-cell-mediated anti-tumour responses appears to be hindered via mechanisms of dysfunction and immunosuppression.

This emerging understanding opens potential therapeutic avenues for treatment of *NF2*-SWN-associated VS, particularly using ICB. Whilst such treatment has revolutionized the management of other solid malignancies, their application in *NF2*-SWN-associated VS remains largely underexplored. Given the nature of the VS TME and the prevalent upregulation of immune checkpoint receptors on infiltrating T cells and their ligands on tumour-associated cells, ICB could potentially reinvigorate dysfunctional T-cell responses and promote tumour control. However, the rational deployment of T-cell-based immunotherapies requires a more detailed understanding of the immune landscape of VS.

Future translational efforts to find treatment for NF2-SWN-associated VS must address several key biological prerequisites. For example, the heterogeneous VS TIL population, their functional states and their organization within the TME must be understood. Furthermore, the response of TILs largely depends on the presence of professional antigen-presenting cells with sufficient co-stimulatory capacities, capable of presenting VS tumour-associated antigens (TAA) to sustain durable anti-tumour responses.^197^ However, both the abundance and functional state of antigen-presenting cell populations and the identity and immunogenicity of relevant VS TAA’s remain poorly defined. Early vaccination approaches, such as VEGFR-directed peptide immunization, demonstrate the feasibility of inducing tumour-specific immune responses in *NF2*-SWN-associated VS. However, the durability and breadth of these responses remain uncertain, particularly given the incomplete characterization of VS TAA’s and the immunosuppressive landscape of the TME. A more detailed understanding of antigen specificity, T-cell functional state and antigen-presenting cell competence will be required to optimize vaccine-based strategies and sustain meaningful clinical benefit.

Collectively, current evidence suggests that effective immunotherapeutic strategies for *NF2*-associated VS are more likely to emerge from rational combination approaches rather than single-agent monotherapy. Particularly promising avenues include strategies cantered on ICB, either through concurrent targeting of multiple immune checkpoints or through integration with agents that inhibit tumour-intrinsic signalling pathways, such as kinases and kinase receptors. In parallel, combinations incorporating immunomodulatory agents directed at myeloid populations may offer opportunities to deplete or reprogramme macrophages and alleviate immunosuppressive features of the TME.

Importantly, the systemic evaluation of such combinations will require mechanistic insight and appropriate preclinical models, alongside biomarker-guided patient stratification based on features such as PD-L1 localization, macrophage abundance and T-cell functional state. Progress in this area will ultimately depend on linking biological understanding of the VS immune microenvironment to clinical meaningful endpoints, including volumetric tumour control and preservation of hearing function.

## Supplementary Material

fcag192_Supplementary_Data

## Data Availability

Data sharing is not applicable to this review article as no new data or analysis was created or performed for this study.
