# Supplementary material for: T-cell-targeted immunotherapy in neurofibromatosis type 2-related vestibular schwannoma: current evidence and future direction
Source: Brain Commun. 2026 May 28;8(3):fcag192. doi: 10.1093/braincomms/fcag192 (PMC13249056; doi:10.1093/braincomms/fcag192)
Supplement: fcag192_Supplementary_Data [file fcag192_supplementary_data.docx]

Supplementary table 1 - Therapeutic agents evaluated in clinical and preclinical studies of vestibular schwannoma.

| Agent | Study / Trial | Model | Target | Outcome |
| --- | --- | --- | --- | --- |
| *Immune checkpoint blockade* | | | | |
| Pembrolizumab | Growth arrest of a refractory schwannoma after anti-PD-1 antibody treatment (S1) | Human | PD-1 | Sustained tumour growth arrest for 30 months following an 18-month anti-PD-1 course (single case report; Level IV evidence) |
| Anti-PD-1 and Bevacizumab | Enhanced tumor control and hearing loss prevention achieved with combined immune checkpoint inhibitor and anti-VEGF therapy in schwannoma model (S2) | Murine orthotopic syngeneic model | VEGF and PD-1 | Bevacizumab enhanced anti-PD-1 efficacy by normalising tumour vasculature. Enhances T-cell and NK cell anti-tumour cytotoxicity and increased tumour control |
| *Immunisation approaches* | | | | |
| VEGFR1-2 peptide vaccine | Phase I/II Study of a Vascular Endothelial Growth Factor Receptor Vaccine in Patients With NF2-Related Schwannomatosis (S3) | Human | VEGFR1-2 | No severe vaccine-related adverse events. 5/13 with assessable hearing improved at 6 months, and 2/13 at 12 months. Partial response in ≥1 schwannoma in 4, minor response in 5 and stable disease in 4 at 2 years. Radiologic response in 9/20 assessable schwannomas. VEGFR1-2-specific cytotoxic lymphocytes induced in 11 patients. |
| *Anti-angiogenic therapies* | | | | |
| Bevacizumab | NCT01207687 | Human | VEGF-A | Completed; ~35–40% achieved hearing response at 6 months; ~32% had ≥20% tumour reduction at 6 months; grade ≥3 AEs reported (notably hypertension). |
|  | Efficacy and safety of bevacizumab for vestibular schwannoma in neurofibromatosis type 2: a systematic review and meta-analysis of treatment outcomes (S4) |  |  | Partial tumour regression in 41%, no change in 47% and progression in 7%. Hearing improvement in 20%, hearing stability in 69% and hearing loss in 6% |
|  | Hearing improvement after bevacizumab in patients with Neurofibromatosis Type 2 (S5) |  |  | 9/10 patients exhibited reduced tumour volume. 6/10 patients exhibited ≥20% reduction in tumour volume. 4/10 exhibited significant hearing response, 2/10 stable hearing and 1/10 progressive hearing loss |
| Axitinib | NCT02129647, NFS-11. Phase II study of axitinib in patients with neurofibromatosis type 2-related Schwannomatosis and progressive vestibular schwannoma (S6). | Human | VEGFR1-3 (primary); also, PDGFR-α/β and c-KIT (off-VEGFR activity) | Completed; 2/10 patients exhibited ≥20% reduction in tumour volume. Most significant response is 53.9% reduction in tumour volume. Hearing responses in 3/9 evaluable patients |
| Sorafenib | Phase 0 trial investigating the intratumoural concentration and activity of sorafenib in neurofibromatosis type 2 (S7). | Human | Multi-kinase inhibitor | Increased macrophage and T-cell infiltration into peripheral schwannomas |
| *Targeted kinase inhibitors* | | | | |
| lapatinib | NCT00863122, Phase II trial of lapatinib in adult and pediatric patients with neurofibromatosis type 2 and progressive vestibular schwannomas (S8) | Human | EGFR, HER2 | 4/17 patients achieved reduction ≥15% tumour volume. 12-month progression free survival of 70.86% and hearing progression free survival of 88.9% |
| Icotinib | NCT02934256, Phase II trial of icotinib in adult patient with neurofibromatosis type 2 and preogressive vestibular schwannoma (S9). | Human | EGFR | 1/10 patients exhibited ≥20% reduction in tumour volume. 3/7 evaluable patients exhibited greater hearing response |
| Crizotinib | NCT04283669 | Human | ALK, ROS1 | Completed; no results posted |
| brigatinib | NCT04374305, Brigatinib in NF2-Related Schwannomatosis with Progressive tumors (S10) | Human | Multi-kinase inhibitor (ALK inhibitor drug class; NF2 mechanism likely ALK-independent) | 23% of all tumours exhibited radiographic response. Meningiomas and non-vestibular schwannomas had the greatest benefit. Reduced annualised growth rates and 35% of eligible patients exhibited hearing improvement. |
| Everolimus / RAD001 | NCT01345136 | Human | mTOR | Terminated |
|  | NCT01490476 |  |  | Completed; varied response, no reduction in tumour volume ≥20%, 4/10 progressed 5/10 exhibited stable disease with a decreased annual VS growth rate |
|  | NCT01880749 |  |  | Completed; No results posted |
|  | NCT01419639 |  |  | Completed; 5.72% reduction in tumour size from baseline |
| Selumetinib | NCT03095248 | Human | MEK1-2 | Terminated |
| *Epigenetic and translational modulators* | | | | |
| AR-42 | NCT02282917 | Human | HDAC | Terminated |
|  | Effect of AR42 in Primary Vestibular Schwannoma Cells and a Xenograft Model of Vestibular Schwannoma (S11) | Primary human vestibular schwannoma cells *in-vitro,*  Cochleovestibular nerve xenograft model |  | Dose-dependent decrease in cell viability (<20% viability reduction at ≥0.75µM. No significant reduction in tumour growth or hearing preservation. |
| *Hormonal modulators* | | | | |
| mifepristone | Computational repositioning and preclinical validation of mifepristone for human vestibular schwannoma (S12) | Primary human VS cell lines and HEI-193 human schwannoma cells | Progesterone and glucocorticoid receptors. | Significantly reduced metabolic activity and proliferation of cell lines. Primary human VS cells were sensitive and normal Schwann cells were left unaffected |
| *Cytokine, Myeloid and inflammatory pathway targeting* | | | | |
| Anti-IL-6 and EGFR blockade | Co-Targeting IL-6 and EGFR signaling for the treatment of schwannomatosis and associated pain (S13) | Patient-derived xenograft murine model | IL-6 and EGFR | Combined agents simultaneously controlled pain and tumour growth. |
| Anti-TNFα | Secreted factors from Human Vestibular Schwannomas can cause cochlear damage (S14) | Human explant cultures | TNFα | Antibody mediated neutralisation of TNFα partially prevented hair cell death in explant cultures. |
| CSF1R blockade | Multiplatform molecular analyses reveal two molecular subgroups of NF2-related schwannomatosis vestibular schwannomas with distinct tumour microenvironment and therapeutic vulnerabilities (S15) | Patient-derived xenograft murine model | CSF1R | CSF1R blockade depleted tumour-associated macrophages and significantly suppressed tumour growth compared to control treatment in immune enriched schwannoma model. |
| ^68^Ga]-Pentixafor (CXCR4-targeted PET ligand) | [^68^Ga]-Pentixafor PET/CT for CXCR4-Mediated Imaging of Vestibular Schwannomas (S16) | Human | CXCR4 | PET/CT scans were visually positive in all cases. SUVmean 3.0 ± 0.3, SUVmax 3.8 ± 0.4; TBRmean 4.0 ± 1.4, TBRmax 5.0 ± 1.7. validation via histology confirmed CXCR4 expression in tumours. |

Supplementary table 1 reference list:

S1. Kouzel Martinez F, Graffeo CS, Carlstrom LP, Link MJ. Growth arrest of a refractory vestibular schwannoma after anti-PD-1 antibody treatment. BMJ Case Rep. 2021 May;14(5):e241834. doi:10.1136/bcr-2021-241834

S2. Lu S, Yin Z, Wu L, Sun Y, Chen J, Wu LMN, et al. Enhanced Tumor Control and Hearing Loss Prevention Achieved with Combined Immune Checkpoint Inhibitor and Anti-VEGF Therapy in Vestibular Schwannoma Model. BioRxiv Prepr Serv Biol. 2024 Dec 29;2024.12.29.630658. doi:10.1101/2024.12.29.630658 PubMed PMID: 39763968; PubMed Central PMCID: PMC11703165.

S3. Tamura R, Yamanobe Y, Fujioka M, Morimoto Y, Fukumura M, Nakaya M, et al. Phase I/II Study of a Vascular Endothelial Growth Factor Receptor Vaccine in Patients With NF2-Related Schwannomatosis. J Clin Oncol. 2024 Jul 20;42(21):2578–87. doi:10.1200/JCO.23.02376

S4. Lu VM, Ravindran K, Graffeo CS, Perry A, Van Gompel JJ, Daniels DJ, et al. Efficacy and safety of bevacizumab for vestibular schwannoma in neurofibromatosis type 2: a systematic review and meta-analysis of treatment outcomes. J Neurooncol. 2019 Sep;144(2):239–48. doi:10.1007/s11060-019-03234-8

S5. Plotkin SR, Stemmer-Rachamimov AO, Barker FG, Halpin C, Padera TP, Tyrrell A, et al. Hearing Improvement after Bevacizumab in Patients with Neurofibromatosis Type 2. N Engl J Med. 2009 Jul 23;361(4):358–67. doi:10.1056/NEJMoa0902579

S6. Garcia MR, Hagiwara M, Yaffe A, Mitchell C, Akshintala S, Nicolaides T, et al. Phase II study of axitinib in patients with *NF2* -related schwannomatosis and progressive vestibular schwannomas. Neuro-Oncol Adv. 2025 Jan 6;7(1):vdaf083. doi:10.1093/noajnl/vdaf083

S7. Ammoun S, Evans DG, Hilton DA, Streeter A, Hayward C, Hanemann CO. Phase 0 trial investigating the intratumoural concentration and activity of sorafenib in neurofibromatosis type 2. J Neurol Neurosurg Psychiatry. 2019 Oct;90(10):1184–7. doi:10.1136/jnnp-2018-319713

S8. Karajannis MA, Legault G, Hagiwara M, Ballas MS, Brown K, Nusbaum AO, et al. Phase II trial of lapatinib in adult and pediatric patients with neurofibromatosis type 2 and progressive vestibular schwannomas. Neuro-Oncol. 2012 Sep;14(9):1163–70. doi:10.1093/neuonc/nos146

S9. Zhao F, Li S wei, Zhang S, Li P, Zhao C, Zhao X bin, et al. Phase II trial of icotinib in adult patients with neurofibromatosis type 2 and progressive vestibular schwannoma. J Neurosurg. 2023 Jun 1;138(6):1680–7. doi:10.3171/2022.9.JNS22699

S10. Plotkin SR, Yohay KH, Nghiemphu PL, Dinh CT, Babovic-Vuksanovic D, Merker VL, et al. Brigatinib in *NF2* -Related Schwannomatosis with Progressive Tumors. N Engl J Med. 2024 Jun 27;390(24):2284–94. doi:10.1056/NEJMoa2400985

S11. Misztal C, Bracho O, Bas E, Estivill M, Ivan ME, Morcos J, et al. Effect of AR42 in Primary Vestibular Schwannoma Cells and a Xenograft Model of Vestibular Schwannoma. Otol Neurotol. 2022 Jul;43(6):694–701. doi:10.1097/MAO.0000000000003556

S12. Sagers JE, Brown AS, Vasilijic S, Lewis RM, Sahin MI, Landegger LD, et al. Computational repositioning and preclinical validation of mifepristone for human vestibular schwannoma. Sci Rep. 2018 Apr 3;8(1):5437. doi:10.1038/s41598-018-23609-7

S13. Yin Z, Wu L, Zhang Y, Sun Y, Chen JW, Subudhi S, et al. Co-Targeting IL-6 and EGFR signaling for the treatment of schwannomatosis and associated pain. BioRxiv Prepr Serv Biol. 2023 Feb 6;2023.02.06.527377. doi:10.1101/2023.02.06.527377 PubMed PMID: 36798353; PubMed Central PMCID: PMC9934519.

S14. Dilwali S, Landegger LD, Soares VYR, Deschler DG, Stankovic KM. Secreted Factors from Human Vestibular Schwannomas Can Cause Cochlear Damage. Sci Rep. 2015 Dec 22;5:18599. doi:10.1038/srep18599 PubMed PMID: 26690506; PubMed Central PMCID: PMC4686978.

S15. Zhao F, Teng XF, Zhang J, Li SW, Wang LM, Zhao HG, et al. Multiplatform molecular analyses reveal two molecular subgroups of NF2-related schwannomatosis vestibular schwannomas with distinct tumour microenvironment and therapeutic vulnerabilities. Acta Neuropathol (Berl). 2025 May 9;149(1):47. doi:10.1007/s00401-025-02883-6

S16. Breun M, Monoranu CM, Kessler AF, Matthies C, Löhr M, Hagemann C, et al. [68Ga]-Pentixafor PET/CT for CXCR4-Mediated Imaging of Vestibular Schwannomas. Front Oncol. 2019 Jun 12;9:503. doi:10.3389/fonc.2019.00503
